# Oral tissue spheroid, organoid, and organ‐on chip microphysiological modeling strategies towards enhanced emulation of health and disease

**DOI:** 10.1002/btm2.70020

**Published:** 2025-04-18

**Authors:** Z. Gouveia, A. Özkan, W. V. Giannobile, J. P. Santerre, D. T. Wu

**Affiliations:** ^1^ Department of Oral Medicine, Infection, and Immunity Harvard School of Dental Medicine Boston USA; ^2^ Faculty of Dentistry University of Toronto Toronto Canada; ^3^ Wyss Institute for Biologically Inspired Engineering Harvard University Boston USA; ^4^ Institute of Biomedical Engineering University of Toronto Toronto Canada; ^5^ Translational Biology and Engineering Program University of Toronto Toronto Canada; ^6^ Harvard John A. Paulson School of Engineering and Applied Sciences Cambridge USA; ^7^ Faculty of Dental Medicine and Oral Health Sciences McGill University Montreal Canada

**Keywords:** microphysiological culture systems, oral health, regenerative medicine, translation, tissue engineering, spheroid, organoid, organ on chip

## Abstract

Diseases and disorders of dental, oral, and craniofacial (DOC) tissues represent a significant global health burden and have been found to have the greatest age‐standardized prevalence and incidence of all reported diseases worldwide. While the application of novel therapies has been suggested to address the different types of oral health diseases, only a limited number of interventional regenerative therapies have been reported to improve clinical therapeutic outcomes. The lack of novel therapies in DOC tissue regeneration may be in part attributed to the highly resource‐intensive translational path from preclinical models to clinical trials. Recently, stakeholders and regulatory agencies have begun to encourage the use of alternative preclinical models using human tissues for testing therapeutic interventions in place of animal models. This advocacy may provide an opportunity to reduce or eliminate animal testing, ultimately limiting resource expenditure and providing a more efficient regulatory pathway for the approval of novel DOC therapies. While the complexity of DOC physiology, defects, and diseases is not effectively recapitulated in traditional 2D or 3D in vitro culture models, the emergence of more sophisticated in vitro models (or so‐called microphysiological systems that include spheroid, organoid and organ on‐chip (OoC) systems) has enabled effective modeling of clinically simulated disease states in several DOC tissue and organ systems. Here, we aim to provide an overview and collective comparison of these microphysiological systems, outline their current uses in DOC research, and identify important gaps in both their utilization and abilities to recapitulate essential features of native oral‐craniofacial physiology, towards enabling the therapeutic performance of de novo interventions targeted at regeneration outcomes in vivo.

**Translational Impact Statement**

DOC biomaterials and regenerative strategies are currently underrepresented in bioengineering and tissue engineering literature—despite the incidence of the disease of such tissues having the greatest age‐standardized prevalence worldwide. The aim of this work is to highlight this subject area through the review of contemporary  microphysiological systems in therapeutic developments and how these emerging technologies may be utilized to improve the therapeutic development pipeline in DOC tissue regeneration.

## 1 INTRODUCTION TO DENTAL ORAL CRANIOFACIAL TISSUES AND DISEASE

The dental, oral, and craniofacial (DOC) complex represents a heterogeneous group of interconnected tissue niches consisting of the dentition, with the periodontium acting to support teeth, and the craniofacial complex that includes other supporting bone, cartilage, ligaments, muscle, as well as peripheral nervous tissue, vasculature, lymphatic vessels, and glands.

Diseases and disorders of DOC tissues represent a significant global burden and have been shown to have the greatest age‐standardized prevalence and incidence worldwide.^1^, ^2^ The annual treatment of oral diseases, independently, has been estimated at >$350B USD in direct costs and represents nearly 5% of global healthcare expenditure. In addition to the direct consequences of DOC disease and disorders that compromise the quality of life and wellbeing of individuals,^3^ the chronic disease state of oral tissues (such as periodontitis) has been increasingly associated with more than 60 systemic diseases, including cardiovascular diseases, diabetes, cancer, pneumonia, inflammatory bowel diseases, obesity, premature birth, and Alzheimer's disease.^4^, ^5^, ^6^, ^7^

Specifically for tissues requiring regenerative interventions to address clinical needs, it has been suggested that novel biological and tissue engineering approaches be further emphasized in the pre‐preclinical model work. Such contemporary therapies are grounded in foundational aspects of tissue engineering and regeneration, including managing the interplay of tissue scaffolds, signaling molecules/growth factors, and/or direct cell delivery for endogenous cell or mediator recruitment. Emerging therapies in DOC include, for example, biomaterial scaffolds that better recapitulate the native architecture of target tissues towards improved healing and integration,^8^, ^9^ the delivery of clinically approved biologics (i.e., rhPDGF‐BB,^10^, ^11^ rhBMP‐2,^12^, ^13^, ^14^ rhFGF‐2,^15^ enamel matrix derivative^16^), that address the inflammatory cascade as well as the repair or regeneration of diseased or damaged DOC tissues,^17^, ^18^, ^19^ and cellular gene therapies^20^, ^21^ that manipulate endogenous progenitors using gene‐loaded vectors to produce pro‐regenerative paracrine factors. Despite promising preclinical evidence with animal studies for many novel biomaterial, biologic and cellular interventions, a limited number of these therapies are available clinically to address the global burden of disease, with only a handful of defined biologics and biomaterials being approved over the last two decades.

The low number of novel therapies for DOC tissue regeneration making it to clinical use can be attributed in part to the high resource‐intensive translational path needed to take an intervention from preclinical animal models to clinical trials; driven mainly by the number of animal tests needed to address regulatory hurdles.^22^ Recently, stakeholders and regulatory bodies have been encouraging the use of alternatives to animal models, specifically human‐derived in vitro models, motivated from both a humanitarian perspective and questions related to their relevance as preclinical models of human disease states.^23^ Several studies have now shown that results for regenerative therapies associated with animal studies may poorly predict therapeutic efficacy in humans, particularly in the case of emerging biologic therapies that investigate gene modification or antigen targeting in human cells.^24^, ^25^ This push for alternative preclinical in vitro models has been directly acknowledged by the US Food and Drug Administration (FDA) Modernization Act of 2021 and the Humane Research and Testing Act (HR 1744). This call may provide an opportunity to reduce or eliminate animal testing, ultimately reducing resource expenditure and providing a more efficient regulatory pathway for the approval of novel DOC therapies—following promising avenues adopted in other clinical fields of tissue regeneration.^26^

Due to the diverse and interconnected nature of DOC tissues, defects and diseases are not effectively recapitulated in traditional 2D or 3D in vitro culture models. The emergence of more sophisticated in vitro models (including spheroid, organoid and organ on‐chip (OoC) systems) has enabled a more effective modeling of clinically representative disease states in several whole tissue and organ systems.^23^ Spheroids are self‐assembled cell aggregates that overcome aspects of the limited cell–cell and cell–ECM interactions associated with traditional culture methods. Organoids feature the same 3D aggregate template of spheroid systems while including complex clusters of organ‐specific cells, which can be more amenable to investigating heterogeneous cell–cell interactions and paracrine signaling or studying tissue organogenesis. On‐chip systems encompass all the features of spheroid and organoid systems while introducing dynamic microfluidics, which may be used to couple multiple cell, tissue, or organ systems to more closely emulate physiological function. Together, such advanced in vitro models (or microphysiological systems) can be designed to provide a high degree of clinical mimicry as they enable the use of health human and pathological cells and their ECM. These parameters can be beneficial in modeling the interconnection of diverse DOC tissues—given that defects and diseases are often made up of a broad etiological origin and present unique cascade pathologies and sequelae.

While there are several reviews that provide detailed analyses for each of the technology and design features associated with the above three individual microphysiological systems independently, when considered for use in DOC tissue engineering,^27^, ^28^, ^29^ the aim in this current review is to build deeper insight onto these reports, and more importantly to provide a comparison of these microphysiological systems to each other, outlining their current uses in DOC research, and identifying current gaps in both their utilization and their ability to recapitulate essential features of native physiology. This is done towards assessing their ability to predict regeneration and therapeutic performance outcomes when applied to humans in vivo. This review will also provide a prospective look on the use of these microphysiological culture systems and their potential to improve the therapeutic development pipeline for DOC tissue regeneration.

## 2 BEYOND STATIC 3D CULTURE—SUMMARY OF CONTEMPORARY IN VITRO SYSTEMS

Prior to animal and preclinical models, static two‐dimensional (2D or monolayer) cell culture, in which cells are cultured on flat and rigid substrates, has been traditionally used to model a number of targeted biological and disease processes in vitro. While static 2D cell culture presents a standardized and cost‐effective platform for initial high‐throughput therapeutic screening, cells cultured in this manner have been shown to poorly represent their in vivo morphology, organization, and behavior. Evidence to support this discrepancy has been described since the 1980s, following the work of Bissel et al. that highlighted the importance of the extracellular matrix (ECM) and microenvironment on the behavior of mammalian cells.^30^ As a result of these findings, it has become widely accepted that essential tissue‐specific architectural, mechanical, and biochemical cues are lost under 2D culture conditions, resulting in non‐predictive and misleading evidence of in vivo responses.^31^, ^32^, ^33^

**FIGURE 1:**
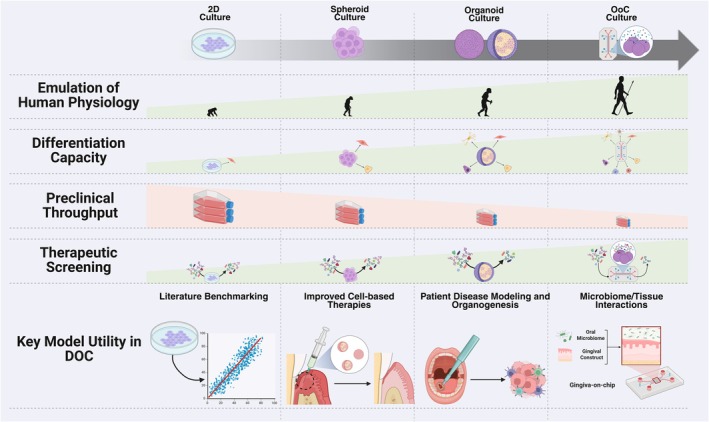
Representative advantages, disadvantages and key utility features of 2D, spheroid, organoid, and organ on chip preclinical model. Green and red are used to represent positive and negative attributes of increasing microphysiological model sophistication. This figure was created with the assistance of www.Biorender.com.

To improve the physiological relevance of cell‐based assays, three‐dimensional (3D) culture methods have been adopted that better model the spatial microenvironment of mammalian cells. Indeed, 3D cultured cells have been shown to maintain more biologically relevant cell–cell and cell–ECM‐like behavior when compared to 2D culture, including more representative cell adhesion and migration, morphogenesis, gene expression, and maintenance of differentiated tissue‐specific phenotypes.^34^ 3D culture may be supported by an ECM‐like support (or scaffold/matrix) or through the induced or natural aggregation of cells in suspension or non‐adherent environments. Different types of contemporary 3D culture systems include spheroid, organoid, and on‐chip models that represent a hierarchy of microphysiological simulation and complexity. A general overview of these model systems is described below and summarized in Figure 1.

### 2.1 Spheroids

Cellular spheroids (or cell aggregates) represent a diverse class of culture systems that have been present since the 1970s.^35^ Spheroids are formed by the spontaneous or forced aggregation of cells, that is followed by the binding of cell surface integrins to ECM (or ECM‐like matrices). Cell–cell contact and the density of aggregates then become regulated by E‐cadherin, which accumulates on the cell surface and may be highly dependent on defined culture conditions, including access to nutrients, oxygen, and growth factors.^36^

Spheroids have become particularly prevalent in tumor modeling, as cell aggregates often contain the same vascular and diffusion limitations of tumors, observed in vivo.^37^ Specifically, multicellular tumor spheroids present greater chemotherapeutic resistance when compared to cancer stem cells in monolayer cultures—a feature that makes them more similar to their in vivo counterparts and thus a more useful model mechanism of chemoresistance.^38^, ^39^ In recent years, the use of spheroids has been expanded to explore several cell types and their related physiologies, including musculoskeletal, nervous, cardiovascular, respiratory, and digestive systems.^40^, ^41^

Spheroids can be formed using several techniques which lead to a predictable and consistent spheroid morphology.^42^ These methods include, for example, the use of liquid overlay, spinner flasks, hanging drop methods, and more recently, being established within individual culture wells for high‐throughput analysis, a topic that has been covered at length elsewhere.^43^, ^44^, ^45^, ^46^

In general, spheroid systems present an *accessible* means to expand culture translation and are amenable to continued exploration into organoid models and on‐chip systems which introduce flow.

### 2.2 Organoids

Organoid models represent a more recent class of cell aggregate microphysiological systems. The key differentiating feature between spheroids and organoids is that organoids contain organ specific cells that may undergo differentiation and display aspects of tissue organogenesis. Organoids have also been shown to facilitate improved differentiation into diverse progenitor cell populations when compared to spheroid culture alone.^47^ In this way, organoid systems may be used to better model whole tissue or organ‐specific architecture, including important multicellular boundaries and junctions that support their specialized function in vivo.^48^, ^49^

Organoids may also be engineered using organ‐specific cells or those directly isolated from patient tissues, allowing for replication of a personalized disease pathology and subsequent therapeutic screening. While providing similar superficial structure to that of spheroids, the formation of engineered multicellular organoids often introduces additional complexities to the aggregate methods described above, attributed to the modulation of co‐culture seeding ratios and selective differentiation of tissue niches. Some organoid systems, however, have shown spontaneous organogenesis in co‐culture conditions that more similarly resemble spheroid formation methodologies, thereby identifying a limitation of these models.^50^ Organoids have also been shown to have the ability to be sustained for long‐term culture and enable effective cryopreservation, showing enhanced genomic and transcriptomic stability when compared to the high mutation rate of progenitor cells and therefore serving as a particularly consistent microphysiological model.^51^, ^52^

In general, organoid systems present an *expanded* microphysiological model that is moving towards translation and the screening of patient specific disease and therapies. The latter feature renders them amenable to continued exploration within on‐chip microfluidic systems.

### 2.3 Organ on‐chip

Advances in high‐resolution microfluidic techniques (such as soft lithography) have given rise to the introduction of cell culture “on‐chip” methods.^53^ Such on‐chip models represent the most recent class of microphysiological culture systems that encompass the features of spheroid and organoid systems while introducing dynamic microfluidics equipped with discrete culture chambers and channels.

The purpose of adding dynamic flow and multichannel design is to provide a model more representative of the physiological exchange of nutrients, elimination of biochemical waste, and mechanical stimuli that govern cell function in vivo. The modulation of waste/nutrient gradients and shear forces, using microfluidics, has been shown to further enhance the differentiation efficiency and fidelity of mesenchymal stem cell organoids in other areas of tissue engineering.^54^ An additional unique feature of on‐chip culture is the ability to effectively culture complex microbiomes with host tissue constructs, which may be co‐cultured for extended periods (up to 5 days) when compared to co‐culture with organoids alone, which can only be maintained for about 6 h.^55^ The study of both mammalian cells in a pathological state with microbiomes is highly relevant to the study of oral diseases and will therefore be a significant aspect of the ongoing discussion in this review article. Further, such capabilities, along with the other benefits of microfluidic culture systems, have expanded the boundaries of what was once thought possible with in vitro models, where such microphysiological systems have been used to develop “functioning” heart, liver, and lung tissues, all of which possess unprecedented similarities to their model organs in vivo.^23^

The design of on‐chip systems and their manufacture is already the subject of an excellent review by Huang et al.^29^ In contrast to other aggregate culture systems, on‐chip methods add considerable cost (equipment, design) and complexity (through the introduction of flow and chip design) to product development workflows, and are currently limited to the availability of chip design for the target tissue type or organ system. Currently, on‐chip methods present the most *complete* means to model in vivo physiology that can provide essential physiological features that may enhance the validation of observations made in aggregate culture.

### 2.4 Towards applications in DOC

The application of these microphysiological culture systems can be useful in modeling the complex nature of the DOC physiology in a systematic manner. Such a strategy may be used to validate therapeutic efficacy or disease in increasingly more bio‐representative models, using each model type to answer specific independent research questions. For example, in a systematic manner, a dental pulp cell spheroid model may be initially used to assess the cytotoxicity of a novel dental adhesive resin.^56^ As an expansion of the spheroid template, an organoid model may utilize a dental pulp/endothelial cell aggregate model, wherein the specific interplay of the pulpal regions with microvasculature and response to cytotoxic agents may be validated.^57^ Further, the modeling of the same dental adhesive resin could also be conducted on‐chip, and may be used to effectively emulate the physiological dentin–pulp interface while modeling the flow state that exists between dental pulp tissues and the oral environment.^58^ Such a system would allow us to study potential diffusive effects on the dilution of cytotoxic agents, as well as their effect on other cells and tissues downstream from where they were introduced. Together, these methods aim to best emulate different physiological tissue states, that may provide answers to what could possibly be observed in vivo. The following text intends to cover examples for the use of these models in DOC (many of which are summarized in Table 1), and to outline how effectively these methods can approach the emulation of the in vivo behavior for DOC tissues.

**TABLE 1:** Summary of microphysiological models utilized within DOC.

Microphysiological	Model utility	Application type	Current DOC gaps
Spheroid	Tissue regeneration	*Regeneration of*: Periodontal ligament^61^, ^62^, ^63^ Gingiva^64^ Dental pulp^65^, ^66^, ^67^, ^68^ Maxillofacial bone^62^, ^70^, ^71^, ^72^, ^73^	Limited applications in screening therapeutics targeted to both dentinal and craniofacial hard tissues.
	Tumor modeling	Oral squamous cell carcinoma (OSCC)^74^, ^75^, ^76^, ^77^, ^78^, ^79^, ^80^, ^81^	
Organoid	Disease modeling and organogenesis	Oral mucositis^84^ OSCC^85^, ^86^, ^88^ Salivary gland organogenesis^87^ Tooth organogenesis^89^, ^90^, ^91^, ^92^	Limited applied cases of organoid use in both therapeutic screening in general and as direct regeneration strategies.
	Therapeutic screening	*Direct or indirect therapeutic effects on*: Dentinal pulp^57^ Taste bud^93^ Salivary gland^94^ OSCC^99^, ^100^	
	Tissue regeneration	*Regeneration of*: Whole tooth^101^, ^102^, ^103^, ^104^, ^105^, ^106^ Salivary gland^87^, ^91^	
Organ on chip	Disease modeling	Oral mucosa/microbial interactions^113^, ^114^, ^115^, ^116^ OSCC^117^, ^119^	Few examples recapitulating craniofacial hard tissues and the functional multi‐domain complex tissues within the periodontium Underutilized in the investigation of direct regenerative oral therapies and biomaterials
	Therapeutic screening	*Treatment of*: Oral biofilms/microbiome^123^, ^124^, ^125^, ^126^, ^127^ *Direct or indirect effects of*: Radiation/chemotherapeutics^128^, ^129^, ^130^, ^131^ Biomaterial interventions^113^, ^115^, ^132^, ^133^, ^134^, ^135^, ^136^, ^137^, ^138^ Multi‐organ and systemic toxicity^139^	

## 3 MODELING DOC TISSUES WITH SPHEROIDS

Spheroids, as discussed above, represent the most accessible class of microphysiological culture systems, given their relatively low resource burden and ease of manufacture. In DOC research, spheroids have found use for modeling aspects of oral soft tissues, including the periodontal ligament and gingival/oral mucosa. Within these applications, spheroids have been utilized as both a tool to improve the efficacy of cell‐based therapies and as a system to provide a more effective therapeutic screening tool.

### 3.1 Improving cell‐based therapies (tissue regeneration)

The delivery of oral progenitor cells has been reported as a useful strategy to aid in tissue remodeling and regeneration.^59^ One current limitation remains the control of the differentiation lineage and capacity of delivered progenitor cells in vivo.^60^ The delivery of cell aggregates/spheroids, in comparison to those delivered from monolayer culture, has been suggested as a method to improve the differentiation capacity of oral progenitor cells. Isolated and cultured periodontal ligament stem cell (PDLSC) populations have been shown to improve their differentiation capacity in spheroid models when compared to standard 2D culture conditions.^61^, ^62^, ^63^ In another example, Zhang et al. reported on a spheroid culture approach to optimize stem cell properties and regenerative processes associated with human gingiva‐derived mesenchymal stem cells (GMSCs) in a chemotherapy‐induced oral mucositis murine model.^64^ The spheroid‐derived GMSCs possessed improved therapeutic efficacy when compared to freely delivered cells, promoting the regeneration of the disrupted epithelial lining in affected tongues, and in reducing body weight loss found in the untreated control group. These findings suggest that 3D spheroid culture enables early stemness preservation, and potentially preconditions GMSCs for enhanced mitigation of oral mucositis.

In addition to oral soft tissues, spheroid systems have been used to improve cell‐based therapies associated with dental and maxillofacial tissues. For dentinal tissues, much attention has been placed on the development of dental pulp stem cell (DPSC) spheroids within the regenerative endodontics field, similar to their utility with multilineage MSCs in other tissue engineering applications.^65^ As with PDLSCs, DPSCs have been shown to present superior multilineage differentiation capacity and differential gene expression in spheroid culture, when compared to monolayer culture.^66^, ^67^, ^68^ The resultant DPSC spheroid systems have also shown a higher expression of both osteo‐ and odontoblastic markers, as well as the enhancement of mineralization and osteogenic potential.^69^

In maxillofacial tissues, spheroids have been utilized to enhance the differentiation capacity of PDLSCs, cortical bone‐derived stem cells (CBSCs), and bone marrow‐derived stem cells (BMSCs) for bone regeneration. When compared to monolayer culture, Moritani et al. found that PDLSC spheroid culture exhibited superior de novo bone formation in a murine defect model.^62^ These findings have been replicated using both cortical bone‐derived stem cells (CBSCs) and bone marrow‐derived stem cells (BMSCs), where spheroid transplantation has resulted in enhanced bone formation and accelerated bone healing in vivo.^70^, ^71^, ^72^ In a spheroid co‐culture model, Zhou et al. found that the co‐delivery of bone marrow stromal cells (BMSCs) and osteocyte‐like MLO‐Y4 cells in the form of spheroids may serve to further enhance scaffold‐free bone regeneration in a murine tooth extraction model.^73^

### 3.2 Therapeutic screening (tumor modeling)

Using DOC spheroid for therapeutic screening has been mainly associated with tumor modeling, given their ability to effectively recapitulate the dense aggregate structure of tumors observed in vivo.^38^, ^39^ In DOC, oral cancer cell lines cultured in spheroids have been shown to have more tumor‐like characteristics when compared to 2D culture.^74^ In addition to more effectively modeling the diffusion limitations of the tumor microenvironment, in comparison to monolayer culture, oral cancer spheroids have been shown to have more physiologically relevant gene and protein expression.^74^, ^75^ The more representative cancer phenotype of oral cancer spheroids has been applied as useful models to screen chemotherapies. For example, Ono et al. showed that exposure to cisplatin and cetuximab elicits significantly different effects on oral cancer cell lines when cultured in spheroid versus monolayer culture conditions.^76^ The effects of both cisplatin and cetuximab were found to have altered chemosensitivity mechanisms in spheroid culture, with the antitumor character attributed to a reduction in cell adhesion and spheroid disruption (dominated by an attenuation of E‐cadherin) and dysregulation of epidermal growth factor receptor expression, that was differentially expressed in each oral cancer cell line and dominated 3D spheroid growth and drug response.

Spheroids have also been used to model oral cancers in xenograft models, which may provide insights into tumor progression and better in vivo screening of chemotherapeutics. Oral squamous cell carcinoma (OCSS), unlike basal cell cancers, is most frequently characterized by the lack of a necrotic tumor core, indicating the maintenance of a pro‐angiogenic niche.^77^, ^78^ To better model this facet of OSCC, Choi et al. utilized an OSCC mouse xenograft model and found that the use of cancer spheroids, when compared to monolayer cells, resulted in significantly larger total tumor volume and enhanced angiogenesis inside the tumor, experiencing little necrosis.^79^ Given its more vascularized structure, only the spheroid‐derived tumors were found to experience significantly reduced volume treatment with cisplatin. To date, spheroid models have found little application in therapeutic screenings for DOC applications, outside of tumor models and chemosensitivity. However, in one such example, Colangelo et al. utilized gingival fibroblast spheroids to assess the efficacy of a polynucleotide‐hyaluronic acid compound to improve oral soft tissue regeneration.^80^ This compound was found to induce an increase in spheroid size and perimeter, and a decrease in spheroid circularity. All the latter characteristics were unable to be observed in previous templated monolayer culture work.^81^

### 3.3 DOC spheroids: summary

Spheroid models have clearly presented significant advantages over monolayer cultures in the analysis of DOC tissues, by introducing more relevant cell–cell and cell–ECM interactions when compared to traditional monolayer cultures. Spheroid methods are currently being used in DOC to improve the regenerative capacity of cell‐based therapies and model oral cancers and chemotherapies. Interestingly, spheroid systems have, to date, seldom been used to screen therapeutics targeted to both dentinal and craniofacial hard tissues. This gap may be representative of the current limitations of spheroids alone, to appropriately represent DOC tissues. For example, while Janjic et al. found that the structure of gingival spheroids was more similar to ex vivo gingival tissues when compared to a monocultured control, they still exhibited significantly different architectural features, including their attachment for collagen and bone‐based substrates and the production of pro‐inflammatory cytokines.^82^ The highly organized and interconnected nature of DOC hard tissues with soft tissue niches perhaps lends itself to be better represented in more comprehensive multi‐cellular systems, as described with organoids below.

## 4 MODELING DOC TISSUES WITH ORGANOIDS

Organoids introduce expanded complexity to traditional spheroid aggregate systems through the addition of organ‐specific multicellular niches. Organoids may be engineered in vitro through the combination of organ‐specific cell types or developed from isolated and differentiated whole donor tissue. In addition to their use in cell‐based therapies and therapeutic screening, organoid systems have found utility in modeling organogenesis and disease progression in DOC research.^83^

### 4.1 Disease modeling and organogenesis

Oral soft tissues are under constant microbial challenge, where their barrier function and stratified structure are essential in maintaining the health of oral tissues. Utilizing multicellular oral mucosal organoids, Pinnock et al. reported significant differences in the response to *P. gingivalis* infection when compared to monolayer cultures of oral epithelial cells.^84^ Intracellular survival and bacterial release of *P. gingivalis* were found to increase in mucosal organoid models (3‐ and 4‐fold, respectively) when compared to monolayer cultures, which can be attributed to the multi‐layered architecture and exfoliation of epithelial cells in these organotypic models. Furthermore, the cytokine profile between infected mucosal organoid models and monolayer cultures was found to be significantly different (particularly for CXCL8 and IL‐6) suggesting an alternative mechanism of cytokine dysregulation by *P. gingivalis* occurs in mucosal organoids, when compared to monolayer culture, which may inform future mechanistic studies in vivo.

In other work, Zhao et al. developed an organoid tongue squamous cell carcinoma (TSCC) model utilizing both CAL27 and cancer‐associated fibroblasts that were immobilized within a decellularized tongue‐derived ECM.^85^ Following implantation in a murine model, the established TSCC organoids presented characteristics resembling clinical TSCC histopathology, including tumor heterogeneity and phenotype, which could serve as an expanded model to carcinomas when compared to spheroid cultures. In another investigation aiming to better model oral SCC, Flashner et al. generated organoids from murine SCC or preneoplastic cells ex vivo through treatment with 4NQO (a known genotoxic agent). These organoids were found to capture some of the essential salient features of ESCC and esophageal preneoplasia and could potentially be leveraged to form isogenic models.^86^

In addition to engineered organoid strategies, in which organotypic cells are combined ex vivo, organoids may be formed through direct isolation and purification from  donor oral tissues. Using this strategy, Yoshimoto et al. established a human salivary‐gland‐derived organoid culture system, capable of modeling salivary gland inflammation.^87^ The model developed induced organoid swelling and provided mechanistic insights into the role of TNF‐alpha on native salivary gland inflammation and dysfunction. Such patient‐ or donor‐derived organoids may yield further insights into patient‐specific disease progression and modeling, towards the development of personalized medicine. For example, in contrast to the purely epithelial cell structure of an OSCC spheroid system, patient biopsied OSCC and formulated organoids contain additional immune, vascular, and stromal associated biomolecular marker character, recapitulating the disease genetically, histologically, and functionally.^88^

A unique feature of organoid aggregate systems is their potential to model organogenesis, which can yield insights into the complex developmental process of DOC tissues. In dental tissues, tooth germ organoids have been developed and used to study previously unknown aspects of human tooth development.^27^ Such investigations into tooth organogenesis have specifically yielded novel insights into signaling pathways (such as Notch and TGF‐beta signaling) that regulate tooth formation in vivo.^89^, ^90^ For oral soft tissues, engineered stem cells have also been utilized to develop a salivary gland organogenesis model, where previously unknown secretory niche factors were identified to be associated with tissue maturation and differentiation from the oral ectoderm.^91^, ^92^

### 4.2 Therapeutic screening

In comparison to spheroid aggregate culture, organoid systems may provide improved utility in screening therapeutics through the presentation of important multicellular boundaries and junctions, as well as patient/donor specificity that is not effectively recapitulated in either spheroid and monolayer cultures.

#### 4.2.1 Improved multi‐cellular architecture

Exploring this utility of organoids to model dental tissues, Xu et al. investigated the differential effects of an endodontic calcium silicate capping agent on both engineered dental pulp organoids and monolayer culture.^57^ In this model, engineered organoids utilized human dental pulp cells (hDPC) and endothelial cells (EC) with (and without) human dental pulp extracellular matrix (hDP‐ECM). This work found that both differential biocompatibility and osteoinductive potential were uniquely expressed in the dental pulp organoid model versus a monolayer culture, although both displayed physiologically relevant mineralization odontogenesis in response to the capping agent. However, this model did not emulate biophysical cues of the dentin–pulp interface, it does represent a promising platform for drug screening and developing restorative materials.

In addition to dentinal tissues, oral soft tissue models have been utilized to investigate the peripheral impact of oral cancer treatments. Guo et al. engineered taste bud organoids to explore the impact of radiation treatment on taste dysfunction.^93^ In exploring an emerging protective therapy, this work found that treatment of taste bud organoids with SIRT1 inhibitors could promote Lgr5+ taste bud stem cell survival and mitigate radiation‐induced tongue mucositis. In another investigation, Serrano‐Martinez et al. investigated the impact of radiotherapy on salivary gland organoids in the induction of hyposalivation‐related xerostomia.^94^ This murine salivary gland organoid model was shown to present long‐term expansion and differentiation capacity, providing a platform to study radiation dose–response curves.

#### 4.2.2 Patient origin and specificity

Patient‐ and donor‐derived organoids have been shown to closely emulate those of the corresponding donor, which has allowed for effective patient‐specific screening of therapeutics outside of DOC.^95^, ^96^, ^97^, ^98^ The use of patient‐derived organoids may even be seen as a method to improve therapeutic screening when compared to animal models, given their ability to present more relevant molecular, cellular, and immunological mechanisms.^23^ The current application of such patient and disease‐specific screening within DOC has focused on the efficacy of chemotherapeutics. In their seminal work related to DOC, Tanaka et al. explored this concept through the investigation of patient‐specific chemotherapeutic sensitivity to patient‐derived head and neck squamous cell carcinoma (HNSCC) organoids.^99^ Formulated HNSCC organoids were found to present similar histological features and drug sensitivity (cisplatin and docetaxel) to those of their genesis tumors, in stark contrast to monolayer cell culture. Sensitivity to chemotherapeutics was also found to be different between HNSCC organoid groups, further recapitulating the characteristics of the original tumors towards the development of precision treatments for HNSCC.

The patient‐heterogeneity of chemotherapeutic efficacy has been further explored by Driehuis et al. utilizing wildtype human oral mucosa organoids in the study of pediatric acute lymphoblastic leukemia.^100^ The developed mucosal organoids effectively recapitulated the multilayered composition, function, and histological characteristics of the native mucosal epithelium and displayed sensitivity to clinically relevant doses of the chemotherapeutic methotrexate and the chemo‐protective drug leucovorin. This work found that the effectiveness of leucovorin in reducing mucosal toxicity differed significantly between organoids derived from different donors, implying a donor‐specific effect and confirming observations of dose dependency observed with the treatment of leucovorin clinically. In a continuation of this work, Driehuis et al. developed 31 head and neck squamous cell carcinoma (HNSCC)‐derived organoid models to recapitulate the genetic and molecular diversity and specificity observed previously.^88^ HNSCC organoid variants were found to have differential responses to conventional chemotherapeutics (including cisplatin, carboplatin, cetuximab), radiotherapy, and targeted drugs that are not normally used in the treatment of patients with HNSCC in vitro. These observations may inspire a personalized approach to the management of HNSCC and expand the repertoire of HNSCC drugs.

### 4.3 Improved cell‐based therapies

Given the hierarchical and heterogeneous nature of DOC tissues, organoid‐based cell therapies have been suggested. In comparison to spheroid‐based cell therapies that are typically utilized for the delivery of stem cell aggregates to promote tissue regeneration, organoid‐based cell therapies employ a higher degree of tissue mimicry and organization, which has great potential in direct whole tissue and organ regeneration in DOC.^101^, ^102^

One such example is the regeneration of de novo dentition utilizing tooth germ organoids. Teeth are complex hierarchical tissues, composed of both hard (enamel, cementum, dentin) and soft (pulp) discrete regions. Tooth germ organoids are formed through tight control of the interactions of dental epithelial and mesenchymal cells achieved through differentiation media and scaffold design. Tsuji et al. were the first to successfully develop ectodermal organs from organoid‐germ culture methods.^103^ The developed tooth germ organoid resulted in the formation of structurally correct teeth in both in vitro organ culture and, following tooth organoid implantation in vivo, which displayed host formation of periodontal ligaments, the penetration of blood vessels, and nerve fibers. This exciting concept for the bioengineered regeneration of whole tooth structure has since been expanded in both canine^104^ and porcine^105^ models. Other tooth germ organoid strategies have expanded on these concepts in using dental pulp stem cells (as opposed to autologous mesenchymal cells) to develop a successful bioengineered dentition.^106^ Current development in this area is focused on scaffold design to guide the development of tooth formation from tooth germ organoids, as bioengineered teeth have yet to match the shape of the natural dentition.

Within the context of oral soft tissues, the application of salivary gland organoids has been explored for its potential use in regenerative therapies. As with natural dentition, salivary glands are particularly difficult to bioengineer given their secretory function, epithelial–mesenchyme interaction, and response to both endogenous and exogenous signals, making them more amenable to an organoid regenerative approach.^87^ Indeed, orthotopically engrafted salivary gland organoids exhibit the features of the nervous system, resulting in full saliva secretion, suggesting that they have great potential for use in the repair and regeneration of salivary glands.^91^

In addition to improved modeling and screening of disease‐ and patient‐specific therapies, patient‐ and donor‐derived organoids have significant potential in direct tissue regeneration strategies.^101^, ^102^ Patient‐isolated organoids benefit from avoiding complications associated with immune rejection and may be more suitable candidates to advance work on the regeneration of multi‐cellular oral tissues in vivo.

### 4.4 DOC organoids: summary

While oral organoids do indeed present an expanded means of oral tissue modeling when compared to both monolayer and spheroid culture, there are still limited applied cases of their use in both therapeutic screening and direct regeneration strategies in DOC. The limited use case of organoids in these applications may be driven by their added complexity, given the need to optimize the microenvironmental conditions for several cell and tissue types simultaneously. Although, these are challenges that have been overcome by other organs in human physiology, and hence it is anticipated that they should be able to be overcome with future studies. Specific examples of challenges that require attention include the consideration of organoid formation (whether engineered or patient derived), which often requires iterative media and culture design (described at length elsewhere^107^), and can add to experimental complexity when compared to both monolayer and spheroid cultures. For example, the culturing of SG organoids relies on the integration and tight control of growth factors (such as BMP‐4, FGF‐7, FGF‐10, and FGF‐2) to modulate their growth and development in culture. Additional complexities arise in the assessment of developed oral organoids, where their engraftment into oral tissues in vivo is frequently used in endpoint evaluations. The latter increases the resource‐intensive nature of oral organoid modeling in isolation. There are also remaining limitations in their physiological character, namely attributed to the need for physiologically relevant mechanical stimuli. Most current methods, applied to modify oral and maxillofacial organoids, rely on simulating the stiffness and architecture of ECM through 3D bioprinting and novel biomaterials as scaffolds. However, precise spatiotemporal regulation and control of self‐organization both need to be attained by utilizing microfluidic devices and further engineered scaffolds.

## 5 MODELING DOC TISSUES ON‐CHIP

On‐chip methods represent the most recent class of microphysiological systems in DOC that encompass the favorable features of both spheroid and organoid culture while utilizing dynamic fluidics and discrete culture chambers. The addition of dynamic flow and associated mechanical cues for in vitro tissues cultured on‐chip has been shown to promote a higher degree of tissue‐emulating features, including improved representation of organ‐specific function and barrier niches.^23^ Tissue niches including the skin and esophagus that share a similar stratified epithelium to that of the oral epithelium have already found application in organ chip models.^108^ Within the context of DOC tissues, on‐chip methods also afford the potential to use unique fluidic interfaces (such as oxygen‐rich environments) to simulate the complex interactions of microorganisms within the oral cavity. Recent reviews have comprehensively described the application of on‐chip models in oral soft tissues,^28^ dentition,^109^ and DOC at large.^29^, ^110^ Here, we specifically highlight the current utility of on‐chip systems in DOC for improving the physiological emulation of disease modeling and therapeutic screening, as well as for enabling the design of regenerative cell therapies.

### 5.1 Environmental stimuli and disease modeling of DOC tissues

Expanding on the concepts of spheroid and organoid culture, on‐chip systems can be utilized to better simulate DOC disease and tissues in vitro, enabled by the introduction of microfluidics and multi‐channel design. The addition of fluidics and multiple channels has enabled more physiologically relevant oral tissue/microbiological interactions and challenges through the introduction of dynamic flow, oxygenated interfaces, and integrated sensor design. Both dynamic flow and oxygenated interfaces can provide a more physiologically relevant environment for studying microbial behavior such as biofilm formation, which is not appropriately emulated or possible to sustain in long‐term static culture. Additionally, the use of on‐chip systems holds the potential to integrate sensor design that, in the case of host tissue/microbiological interactions, has been utilized to assess the integrity and barrier function of epithelial tissues in vitro.^111^, ^112^

In DOC, as an expansion of oral mucosal organoids, oral mucosa on‐chip models have been developed to explore the microbiological impact on oral soft tissues towards a better understanding of mucositis and periodontitis. For example, Rahimi et al. developed a gingival mucosa on‐chip system to investigate the impact of oral bacteria *Streptococcus mutans* (one of the primary etiological agents of dental caries).^113^ This initial system consisted of a gingival fibroblast‐laden collagen hydrogel with an external keratinocyte layer assembled in a three‐channel microfluidic chip. This model produced apical side channels capable of maintaining stability over long‐term culture under physiologically relevant flow and exposure to *S. mutans* challenge. As an expansion of this concept, Jin et al. explored the development of a highly vascularized gingival epithelial barrier on‐chip model.^114^ This model was shown to recapitulate the barrier function and complex organ‐level cascade of responses in human gingival tissue following exposure to bacterial lipopolysaccharide, serving as a potential platform to investigate the progression of periodontal disease. More recently, Sriram et al. developed an alternative microfluidic system that integrated an air‐liquid interface into the development of gingival models on‐chip.^115^, ^116^ Sriram's group has shown that this on‐chip platform can enable representative mucosal matrices, improved epithelial morphogenesis, and barrier features that are stable over long‐term culture. The latter has been used in the investigation of oral mucosal ulcers and co‐culture systems with relevant oral microbes.

Through the addition of flow, on‐chip models have also allowed for the modulation of physical stimuli that are associated with the mechanical and functional features of oral tissues. Le et al. developed an oral mucosa on‐chip platform to investigate the impact of mechanical stimuli on epithelial barrier function, finding that sub‐epithelial tissue stiffness significantly modulates epithelial barrier function.^117^ The modulation of physical cues on‐chip (such as stiffness and shear flow) may also be useful in modeling the severity of OSCC, where the disease state has been observed to result in different mechanical properties in vivo.^118^ OSCC‐on‐chip models have the potential to modulate these and other factors, while integrating the different cell/tissue types to help expand our understanding of the metastatic cascade. For example, using a microfluidic approach, Lugo‐Cintron et al. found that lymphatic vessels exposed to tumor‐derived fibroblasts from patients induced sprouting, altered vessel permeability, and increased the expression of pro‐lymphangiogenic genes in a patient‐specific manner, offering insights into the dysregulated pathways leading to lymphangiogenesis.^119^ Such a platform can provide an essential platform to study rare cancers and patient‐specific comorbidities.

### 5.2 Improved therapeutic screening and systemic toxicity

Given their enhanced degree of tissue and organ emulation, many applications of on‐chip culture systems have investigated their use as therapeutic screening platforms.^120^ As an extension of the enhanced clinical relevance for patient‐derived organoids, the integration of such models on‐chip allows for the modeling of more relevant drug perfusion kinetics, boundary effects, and affords the potential for long‐term culture.^121^

#### 5.2.1 Modulating microbes

As oral microbiome dysbiosis is particularly pernicious and patient specific, on‐chip models have been extensively used to study their behavior.^122^ On‐chip methods have uniquely enabled the direct dynamic study of oral biofilms, where in the work of Nance et al., they utilized microfluidics to develop one of the first oral biofilm on‐chip systems.^123^ Using isolates from whole human saliva, this model specifically recapitulated the composition and architecture of native dental plaque biofilm in vitro, which allowed for multi‐species screening of a quaternary ammonium‐based mouthwash. Similar dental biofilm strategies have been developed using on‐chip platforms aiming to increase throughput,^124^ modified flow regimes,^125^ biofilm visualization,^126^ and resolution of inter‐species and host immune interactions^127^ toward improved development and screening of plaque‐modulating and oral care products and therapies.

#### 5.2.2 Radiation and chemotherapeutic screening

The ability of on‐chip culture platforms to provide a specific tissue niche (as described earlier) makes them useful in the screening of radiative and drug‐based cancer therapies in DOC. Both the radiosensitivity of cancerous and peripheral DOC cell types have been explored in on‐chip models. Kennedy et al. developed a unique tumor‐on‐chip platform to explore the specific effect of radiation treatment on patient tumors cultured ex vivo.^128^ In an expanded model, Song et al. utilized a salivary gland on‐chip model to investigate salivary gland dysfunction following radiation therapy.^129^ In this work, as opposed to organoid culture alone, the engineered chip design enabled long‐term culture, the study of autocrine/paracrine conditioning, and in situ imaging, which allowed for the screening of a radioprotective compound on salivary gland function. Recently, an oral mucositis model was developed to evaluate the peripheral effects of combined radiation and chemotherapeutic treatment on the barrier function of oral mucosal tissue.^130^ This model effectively recapitulated the epithelial damage, ulceration, and cytotoxicity that occurs in mucosal tissue following cancer treatment. This allowed the process to be monitored in situ and has since been used to investigate the impact of recombinant human keratinocyte growth factor in mitigating treatment‐induced mucositis.^131^

#### 5.2.3 Biomaterial interventions

On‐chip methods allow for an expanded means of modeling biomaterial interactions. While direct surface interactions can be seen as effectively modeled in static culture systems, the effect of degradable or leachable materials is best represented in continuous culture.

In DOC, the aformentioned facet has been particularly useful in modeling dental restorative materials, which have been shown to have several cyto‐ and genotoxic leachable elements.^132^, ^133^ Such dental restorative materials have been explored on emerging tooth on‐chip models.^109^ Seminal tooth on‐chip models have recently been developed by Hadjichristou et al. and Bertassoni et al. in parallel.^58^, ^134^, ^135^, ^136^ Both models encompass functional dentin/pulp analogs and have been used as advanced cytotoxicity assessment tools for dental restorative materials including cements, resin adhesives, and bonding etchants. Such tooth on‐chip systems may also be instrumental in elucidating specific aspects of dental restorative failure. Bertassoni et al. found enhanced gelatinolytic activity in the model hybrid layer (HL) in their on‐chip model, which suggests that dental pulp cells may contribute to the proteolytic activity in the HL more than endogenous proteases, which may contribute to restorative failure in vivo.^58^ Hadjichristou et al., in their investigation of cements and adhesives, elucidated combinatorial effects of restorative materials with lipopolysaccharide (LPS) on odontogenesis, angiogenesis, and cell viability.^134^

In addition to the effects of restorative materials directly on dentition, the peripheral effects of leachables on DOC tissues have been explored using on‐chip models. For example, using a mucosa on‐chip model, Rahimi et al. investigated the effect of a leachable restorative monomer (HEMA) on mucosal tissues.^113^ Exposure to HEMA was found to lower mucosal cell viability and transepithelial electrical resistance. Hu et al., in their preparation of their own tooth on‐chip model, compared the cytotoxic effect of silver diamine treatments (SDF) to both tooth and gingival epithelium analogs.^137^ This study found SDF to effectively penetrate dentin and elicit an extreme cytotoxic effect on dental pulp cells in the dentin/pulp chip and exhibit histologically similar toxicity in gingival epithelium equivalents to that found in vivo. Other investigations by Sriram's group have found similar negative peripheral effects of oral restorative rinses on gingival epithelial tissue on‐chip.^115^

Outside of dental restorative assessment, an emerging on‐chip biomaterial strategy has been developed to investigate dental implants. Using their novel dental implant on‐chip model, Dhall et al. investigated the impact of implant architecture multiplexed with bacterial challenge on the integration of periodontal cells.^138^ Using photobiomodulation therapy, the implant epithelial layer was effectively protected from recurrent bacterial challenge and exhibited minimal cellular damage. Such a multiplexed system may provide a more physiologically relevant model to study host‐biomaterial interaction in DOC.

#### 5.2.4 Multi‐organ and systemic toxicity

One unique feature of on‐chip models is the potential to connect multiple organ or tissue systems together. DOC soft tissues, such as the oral mucosa, represent a key barrier in preventing the oral exposure to leachables or toxins from circulatory access and accumulation in peripheral organ systems. Additionally, oral exposure to toxins (such as metals) is known to activate the immune system, which can lead to peripheral effects including skin inflammation. To study this effect, Koning et al. developed a novel gingival and skin multi‐organ on‐chip system for evaluating nickel leachables from dental restorative materials.^139^ This system utilized reconstructed human gingiva and dermal chambers with integrated MUTZ‐3‐derived Langerhans cells (MUTZ‐LC) to investigate localized immune events involved in tolerance, sensitization, and elicitation of contact allergy. While nickel exposure was shown to have no effect on gingival tissues, dermal chambers were found to have increased activation of MUTZ‐LC and sensitization.

### 5.3 DOC on‐chip: summary

Currently, OoC systems represent the most effective means of emulating organ and tissue function in vitro, enabled through the integration of microfluidics, multi‐fluid interfaces, multi‐chamber design, and in situ monitoring. Within DOC, OoC systems are emerging, and there are now some examples that have been utilized to better model environmental stimuli, tissue disease states, therapeutic efficacy, and systemic toxicity. Currently, OoC platforms have had limited application in recapitulating craniofacial hard tissues and functional multi‐domain complex tissues within the periodontium. While bone on‐chip platforms are currently under development,^140^ such systems face challenges associated with bone organogenesis on‐chip, which requires the application of complex mechanical stimuli (in excess of that provided by shear flow) and an appropriate bone microenvironment. The ability to model bony tissue and hard tissue interfaces on‐chip may allow for better investigations into complex DOC disease progression, such as periodontitis. In addition to the lack of an available hard tissue platform, DOC chip platforms are currently under‐utilized in the investigation of direct regenerative oral therapies and biomaterials. The modeling of FDA‐approved biologics (e.g. rhPDGF‐BB) or biomaterials (e.g. collagen matrix) using a relevant soft tissue platform (such as a mucosa on‐chip) could provide an effective means of validating an in vitro system with clinical results.

## 6 MICROPHYSIOLOGICAL SYSTEMS AS PRECLINICAL MODELS IN DOC

Interest in oral health and disease has expanded in recent years,^141^ resulting in enhanced research efforts and trickle‐down methodologies from other important areas of biomedicine, including more sophisticated microphysiological in vitro systems. Together, spheroid, organoid, and on‐chip systems represent useful models to better recapitulate DOC tissues in vitro when compared to conventional 2D culture. Based on their enhanced emulation, OoC platforms present the most likely endpoint in vitro system, capable of replacing or limiting the use of animals as preclinical models, which could significantly reduce resource burden and improve the DOC development pipeline. However, limitations and challenges still need to be addressed, both within DOC‐OoC and OoC methods at large, before their adoption as alternative preclinical models becomes a commonly adopted practice.

Within DOC, there are currently few examples of OoC systems that aim to simulate the multifaceted nature of human disease and progression. For example, few studies within DOC have sought to simulate human immune function on‐chip, which is a feature already prevalent in other OoC platforms that presents a significant advantage over preclinical evaluations utilizing immuno‐compromised vertebrates.^67^, ^142^ Additionally, there are few examples within DOC that aim to elucidate connected disease states—such as the connection of oral pathogens with oral cancer tumorigenesis,^143^ and the potential impact of the microbiome to protect (or exacerbate) radiation‐induced injuries, previously found in intestinal models.^144^ Despite oral disease and disorders increasingly being associated with systemic diseases, there are currently limited examples that connect DOC organ chips in series with other OoCs towards emulating an organ system or “body”‐on chip that have already found applications in other OoC configurations.^145^, ^146^ Outside of DOC, OoC systems are currently limited by standardization and in vivo validation. Standardization is now being addressed through the commercialization of platform microfluidic culture chips (such as those produced by Emulate™ and others), which stand to continually improve both reproducibility and the standardization of OoC findings. Additionally, for the complete replacement of preclinical animal models, OoC systems will need to not only display enhanced tissue homology to the target niche in vivo, but also possess the potential to better predict human clinical performance in vitro when compared to animal models. As regulatory bodies have begun to encourage the use of OoC platforms in preclinical data, this facet will receive increased attention in the coming years.

**FIGURE 2:**
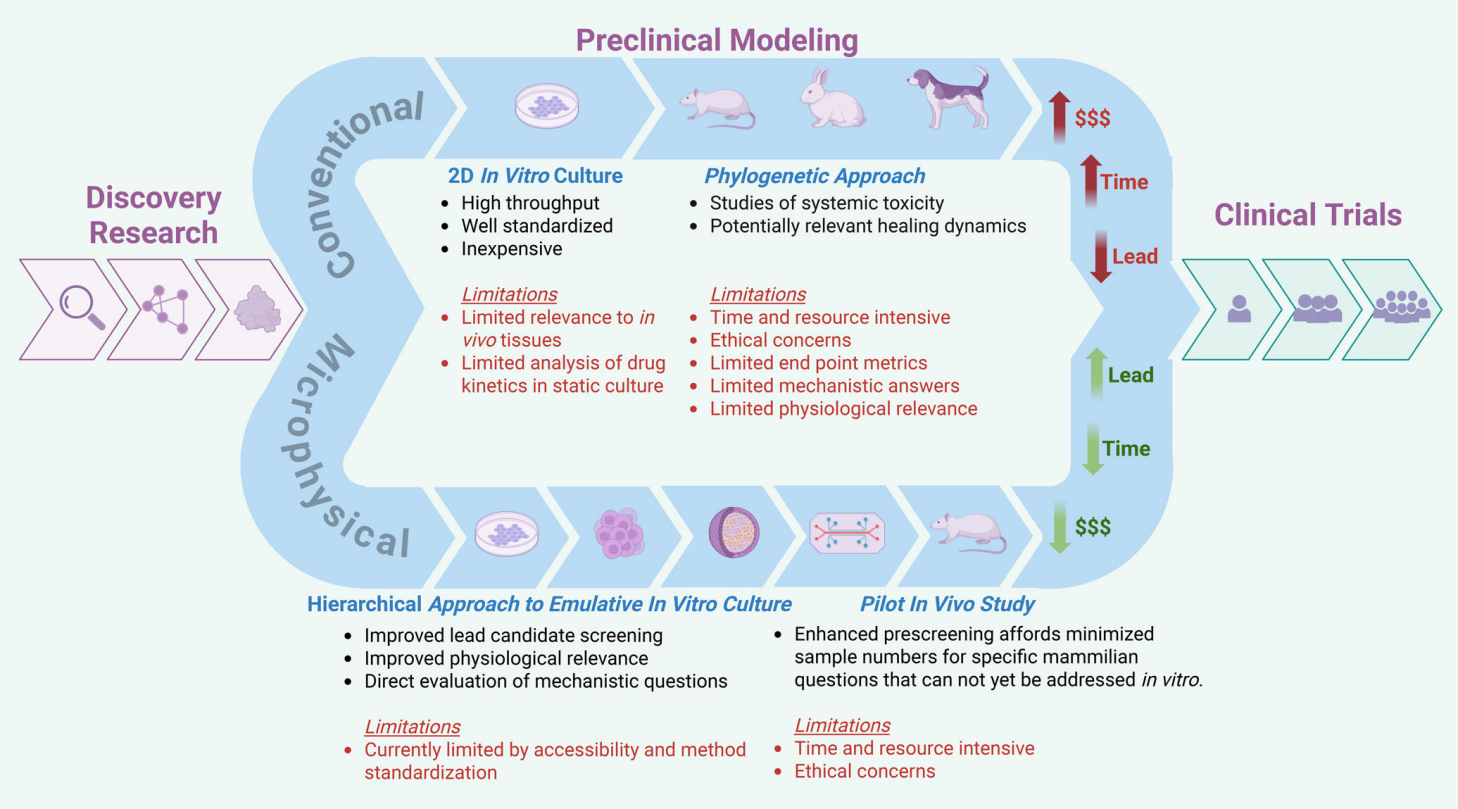
Traditional versus alternative preclinical modeling in DOC therapeutic development. This figure was created with the assistance of www.Biorender.com.

While complete elimination of animals, as preclinical models, may not be possible as of yet, sophisticated microphysiological systems may be employed in an alternative development pipeline towards both reducing animal modeling and potentially improving clinical translation of DOC therapies (Figure 2). Conventional preclinical models in DOC have utilized animals extensively, being present in nearly 40% of recent publications in relevant DOC literature.^147^ Model choice in DOC has developed over the past 50 years,^147^, ^148^ with the species distribution of preclinical animals now consisting of smaller mammals (such as rats and mice) more frequently, which has served to address ethical, cost, and supply considerations. The use of such small animals, however, is hampered by their differences in anatomy and healing, when compared to larger mammals. For this reason, methods within DOC research have always considered phylogeny throughout in vivo studies, where therapeutic validation is conducted sequentially in larger mammals towards increased physiological relevance. In addition to the complexity of multi‐animal studies, in vivo animal models are often unable to recapitulate human disease states such as inflammation,^149^ potentially leading to false positive or negative results for candidate therapeutics to enter clinical trials.

In the alternative development pipeline proposed, traditional monolayer in vitro evaluations are suggested to be followed by increasingly emulative microphysiological models prior to final therapy validation in vivo. This might serve to represent an in vitro analogue of the current phylogenetic approach used in preclinical in vivo models. Employing intermediate evaluations of increasing physiological relevance in vitro, prior to animal modeling, could stand to increase screening efficacy and efficiency, yield better insights into therapeutic mechanisms, and reduce the total number of animal studies. In this scheme, an in vivo model may be used to answer specific questions related to mammalian physiology that cannot be met by current in vitro methods, with significantly reduced sample size and experimental complexity.

Costs require significant consideration in the preclinical modeling phase for the development of novel therapies in DOC, which may account for >40% of total translational costs.^150^, ^151^, ^152^ In the context of publicly funded research, the replacement of one large animal model from preclinical development alone could reduce resource expenditure significantly. In 2024, the National Institute of Health—National Institute of Dental and Craniofacial Research (NIH‐NIDCR) supported a total of 146 first‐year preclinical DOC studies with an average value of $277,000 USD.^153^ Using a conservative estimate of animal maintenance costs alone, a single small preclinical canine study, frequently used to model peri‐implantitis, may cost in excess of $120,000 USD, potentially representing more than 43% of total study funding.^154^ The use of alternative and more relevant sophisticated in vitro models, such as DOC‐OoCs, may reduce the cost of preclinical evaluations, while enabling additional valuable information to be acquired. In screening multiple therapeutic candidates, it has been estimated that OoC models could result in more than a ten‐fold reduction in cost and a four‐fold reduction in study time compared to screening in equivalent large animal models.^155^ In addition, the cost and time reduction of preclinical studies stand to empower researchers in developing economies and in regions with stringent or restrictive animal testing policies to translate benchtop discoveries to the clinical arena.

## 7 CONCLUSIONS

Diseases and disorders of the dental, oral, and craniofacial (DOC) tissues represent a significant global burden and have been shown to have the greatest age‐standardized prevalence and incidence worldwide. While the application of novel therapies has been suggested to address this global burden, a limited number of such regenerative therapies are available clinically to contribute to improving therapeutic outcomes, with only a handful of therapies translated into the clinic over the past 10 years. The lack of novel therapies in DOC tissue regeneration may be attributed to the highly resource‐intensive translational path from preclinical models to clinical trials, driven mainly by extensive testing in animal models to address regulatory hurdles. Recently, stakeholders and regulatory bodies (including the US Food and Drug Administration) have begun to encourage the use of alternatives to animal models, motivated by both a humanitarian perspective and questions about their relevance in preclinical models.

This may provide an opportunity to reduce or eliminate animal testing, ultimately reducing resource expenditure and providing a more efficient regulatory pathway for the approval of novel DOC therapies. As the DOC complex represents a diverse group of interconnected tissue niches, defects and diseases are not effectively recapitulated in traditional 2D or 3D in vitro culture models. However, the emergence of more sophisticated in vitro models (or so‐called microphysiological systems that include spheroid, organoid and organ on‐chip systems) has enabled more effective modeling of DOC disease and regeneration, through enhanced emulation of DOC physiology. While such models are unable to completely replace animal use in preclinical models, an alternative translational pipeline using a pseudo‐phylogenetic approach may improve translational efficiency through enhanced lead candidate screening and by providing a mechanistic understanding of therapeutics on patient‐derived tissues. As OoC platforms within DOC progressively enhance the emulation of human immune and multi‐organ function, they may serve as future animal alternatives for applications towards efficient therapeutic validation and translation into the clinical arena. Together, the use of an in vitro ‘phylogenetic’ approach could reduce the resource burden and potentially improve the success of lead candidate efficacy in clinical trials.

## AUTHOR CONTRIBUTIONS

**Zach Gouveia:** Writing – original draft; conceptualization. **Alican Ozcan:** Writing – review and editing. **William Giannobile:** Writing – original draft; conceptualization. **Paul Santerre:** Writing – review and editing; conceptualization; funding acquisition. **David Wu:** Writing – original draft; conceptualization; funding acquisition.

## CONFLICT OF INTEREST STATEMENT

The authors have no conflicts of interest to declare.

## Data Availability

The data that support the findings of this study are available from the corresponding author upon reasonable request.
